# The Electronic Surviving Cancer Competently Intervention Program—a Psychosocial Digital Health Intervention for English- and Spanish-Speaking Parents of Children With Cancer: Protocol for Randomized Controlled Trial

**DOI:** 10.2196/46339

**Published:** 2023-06-02

**Authors:** Kimberly S Canter, Lee Ritterband, David R Freyer, Martha A Askins, Laura Bava, Caitlyn Loucas, Kamyar Arasteh, Wen You, Anne E Kazak

**Affiliations:** 1 Center for Healthcare Delivery Science Nemours Children's Health Wilmington, DE United States; 2 Center for Behavioral Health and Technology University of Virginia Charlottesville, VA United States; 3 Cancer and Blood Disease Institute Children's Hospital Los Angeles Los Angeles, CA United States; 4 The University of Texas MD Anderson Cancer Center Houston, TX United States; 5 Division of Behavioral Health Nemours Children's Health Wilmington, DE United States; 6 Department of Public Health Sciences UVA Comprehensive Cancer Center University of Virginia Charlottesville, VA United States

**Keywords:** pediatric cancer, digital health, parents, caregivers, psychosocial intervention, family systems, cultural and linguistic adaptation

## Abstract

**Background:**

The psychosocial needs and risks of children with cancer and their families are well-documented including increased risk of parental distress, posttraumatic stress, and anxiety. There is a critical need to provide evidence-based psychosocial care to parents and caregivers of children with cancer. Digital health interventions are important to address many barriers to in-person intervention delivery but are not widely used in pediatric psychosocial cancer care. The COVID-19 pandemic has reinforced the need for flexible, acceptable, and accessible psychosocial digital health interventions. The Electronic Surviving Cancer Competently Intervention Program (eSCCIP) is an innovative digital health intervention for parents and caregivers of children with cancer, delivered through a combination of self-guided web-based content and supplemented by 3 telehealth follow-up sessions with a trained telehealth guide. A Spanish language adaptation of eSCCIP, El Programa Electronico de Intervencion para Superar Cancer Competentemente (eSCCIP-SP), has been developed. The self-guided web-based cores of eSCCIP/eSCCIP-SP are a mix of didactic video content, multifamily video discussion groups featuring parents of children with cancer, and hands-on web-based activities.

**Objective:**

The objective of this study is to test eSCCIP/eSCCIP-SP in a multisite randomized controlled trial, compared to an internet-based education control condition consisting of information specifically focused on concerns relevant to parents and caregivers of children with cancer.

**Methods:**

Using a randomized controlled clinical trial design, 350 eligible parents and caregivers of children with cancer will be randomly assigned to the intervention (eSCCIP/eSCCIP-SP) or an education control condition. Data will be collected at 3 time points: preintervention (prior to randomization), immediately post intervention (after 6 weeks), and at a 3-month follow-up (from baseline). Participants randomized to either condition will receive study material (eSCCIP/eSCCIP-SP intervention or education control website) in English or Spanish, based on the primary language spoken in the home and participant preference.

**Results:**

The primary study end point is a reduction in acute distress from baseline to postintervention, with secondary end points focused on reductions in symptoms of posttraumatic stress and anxiety, and improvements in coping self-efficacy and cognitive coping. An additional exploratory aim will be focused on implementation strategies and potential costs and cost-savings of eSCCIP/eSCCIP-SP, laying the groundwork for future trials focused on dissemination and implementation, stepped-care models, and intervention refinement.

**Conclusions:**

This trial will provide necessary data to evaluate the efficacy of eSCCIP/eSCCIP-SP. This intervention has the potential to be an easily scalable and highly impactful psychosocial treatment option for parents and caregivers of children with cancer.

**Trial Registration:**

ClinicalTrials.gov NCT05294302; https://clinicaltrials.gov/ct2/show/NCT05294302

**International Registered Report Identifier (IRRID):**

PRR1-10.2196/46339

## Introduction

### Background

Parents and caregivers of children with cancer are at risk for negative psychosocial sequelae following their child’s cancer diagnosis [1-3]; yet, few evidence-based options exist for improving psychosocial outcomes. This is particularly discouraging because the Standards for Psychosocial Care for Children with Cancer and Their Families specifically recommend that parents and caregivers of children with cancer have access to such interventions to decrease the risk of adverse psychosocial outcomes, including acute distress, posttraumatic stress symptoms (PTSSs), anxiety, depression, and decreased family functioning [1,4]. Parent, child, and family-level functioning are closely linked, and parent distress is linked to impaired family functioning and child maladjustment across the cancer trajectory [2,5-8]. Providing evidence-based psychosocial support to parents and caregivers of children with cancer is a clear, actionable strategy to prevent adverse outcomes and improve individual and family functioning following a pediatric cancer diagnosis [9,10].

Individuals identifying as Latinx represent the largest minority group in the United States and are also the fastest-growing segment of the population in the United States, representing 18% of the total population and contributing over half (52%) of the population’s growth between 2010 and 2019 [11]. Parents and caregivers of children with cancer, who are of Latinx descent, are at especially high risk for negative psychosocial sequelae because they are overrepresented in the United States in terms of being affected by multiple, negative, systemic, and sociodemographic factors, including low socioeconomic status and limited English proficiency [12]. Socioeconomic status has been found to mediate survival outcomes in pediatric cancer, with worse outcomes for Latinx and racially diverse children [13-15]. Moreover, parents and caregivers of children with cancer of Latinx descent often report high levels of distress following their child’s cancer diagnosis [12,13]. Although there is some evidence that culturally adapted interventions are effective in improving health and well-being outcomes for these populations [16-19], cultural and language adaptations of psychosocial evidence-based interventions for the Latinx population are scarce [1,12,20]. As the Latinx population in the United States continues to grow, there is an urgent need to provide evidence-based psychosocial care to this at-risk subset of parents and caregivers of children with cancer.

Access to technology and the internet is widespread in the United States and is often used to seek health care information and support [21,22]. Latinx and non-Latinx populations report internet use at comparably high levels, with recent data showing that 93% of White adults, 91% of Black adults, and 95% of Hispanic adults use the internet [23]. Trends in internet usage over time have demonstrated that the “digital divide” in the United States is shrinking across race and ethnicity, so the need for psychosocial interventions that adapt to this new reality is critical.

### Intervention Development and Adaptation

**Figure 1:**
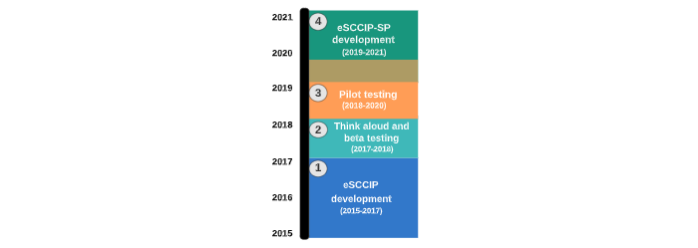
Overview of eSCCIP/eSCCIP-SP development. eSCCIP: Electronic Surviving Cancer Competently Intervention Program; eSCCIP-SP: El Programa Electronico de Intervencion para Superar Cancer Competentemente.

Several studies have laid the groundwork for the proposed randomized controlled trial (RCT). The Electronic Surviving Cancer Competently Intervention Program (eSCCIP) was developed as a brief, psychosocial digital health intervention for parents and caregivers of children with cancer to reduce negative psychosocial sequelae following pediatric cancer diagnosis and improve family functioning [24]. The theory and content of eSCCIP were adapted from 2 prior in-person interventions for parents and caregivers of children with cancer: the Surviving Cancer Competently Intervention Program [25] and the Surviving Cancer Competently Intervention Program—Newly Diagnosed [26]. To provide parents and caregivers of children with cancer with the accessibility and flexibility of a digital health intervention, eSCCIP was initially developed in 2015 using best practice methodology from the digital health literature. In addition to its foundation in both cognitive behavioral and family systems principles, eSCCIP is guided by the Ritterband Behavior Change model for internet interventions, which explains change as occurring through a pattern of unique, nonlinear interactions in 9 steps, including the users, the environment, and the web-based tool [27]. eSCCIP is further guided by the Pediatric Preventative Health Model [28,29], a social ecological model conceptualizing how families should receive psychosocial support based on individual needs, strengths, and risk factors. Following the conceptualization and initial development of eSCCIP, the intervention was refined using think aloud usability testing, collaboration with community stakeholders, and small-scale beta testing [24]. Recent preliminary studies evaluating the eSCCIP intervention have demonstrated high acceptability and feasibility, as well as preliminary evidence of efficacy [30]. Additional revisions and iterations have been made to eSCCIP to modernize and scale up for a larger RCT. To fill a critical need in the field, a Spanish-language adaptation of eSCCIP, El Programa Electronico de intervencion para Superar Cancer Competentemente (eSCCIP-SP) [31], was also developed using best practices for cultural and linguistic intervention development [32]. Refer to Figure 1 for a visual overview of the eSCCIP/eSCCIP-SP work to date.

### Study Objectives

The purpose of this study is to test eSCCIP/eSCCIP-SP in a multisite RCT to build off existent pilot work and establish a strong base for the efficacy of the intervention. With the exception of the present intervention and a web-based adaptation of the Bright IDEAS Maternal Problem-Solving Skills Training Intervention [16,33,34], we are not aware of any additional psychosocial digital health interventions for parents and caregivers of children with cancer, so establishing a strong evidence base for this intervention is both timely and imperative in addressing the psychosocial needs in this population. The specific aims of this study are as follows:

1. Specific aim 1 evaluates the efficacy of eSCCIP/eSCCIP-SP in a multisite RCT comparing eSCCIP/eSCCIP-SP with an education control website in “real-world” clinical settings. The primary hypothesis is that eSCCIP/eSCCIP-SP will be more efficacious than an education control in decreasing acute distress post intervention. A secondary hypothesis is that eSCCIP/eSCCIP-SP will be more efficacious than an education control in decreasing PTSS and anxiety and improving coping self-efficacy and use of positive cognitive coping strategies post intervention.
2. Specific aim 2 describes the patterns of user engagement and identifies clinical and psychosocial factors associated with eSCCIP/eSCCIP-SP treatment gains. A component of this aim is to identify clinical, demographic, and psychosocial protective factors and risks predictive of maximum eSCCIP/eSCCIP-SP participation benefit. Potential differences in outcomes for English-speaking and Spanish-speaking parents and caregivers of children with cancer will also be explored.
3. Specific aim 3 explores preliminary eSCCIP/eSCCIP-SP implementation strategies and costs and cost savings. We will conduct qualitative interviews with oncology health care providers (psychosocial and medical/nursing staff) and intervention participants using the Consolidated Framework for Implementation Research (CFIR) paradigm to identify barriers and facilitators of eSCCIP/eSCCIP-SP uptake and completion. We will also estimate the costs and potential cost savings of delivering eSCCIP/eSCCIP-SP.

## Methods

### Ethics Approval

The MD Anderson Cancer Center Office of Human Subjects Protection serves as the central institutional review board for this study. Study procedures have been approved (protocol # 2021-0988).

### Study Design

This study is a multisite RCT that will enroll participants at 3 geographically diverse pediatric hospitals, which are subsequently anonymized as sites A, B, and C. Participants will be randomized to one of two treatment conditions: (1) eSCCIP/eSCCIP-SP (depending on primary language or preference) or (2) education control.

### Randomization

Randomization will be conducted at the family level according to a 1:1 ratio between groups, in permuted blocks of 6, stratified by site and language preference. The study data analyst at site A will use an Excel (Microsoft Corp) spreadsheet with a randomization scheme created by the study biostatistician to randomize families. This spreadsheet will be inaccessible to any members of the study team aside from the data analyst. It is not possible for study telehealth guides to be blinded to the intervention condition because of the in-person contact required for eSCCIP/eSCCIP-SP; however, study investigators and research coordinators (RCs) not involved in intervention delivery will not have access to random assignment information. After randomization, participants will be instructed on the procedures within their assigned group. All outcome measures will be completed directly on the web by study participants, further reducing the risk of bias.

### Participants

Participants will be parents or caregivers of a child with cancer who speak English or Spanish between ages 0 and 18 years. Parents and caregivers of children with cancer must have access to the internet (including access exclusively via cellular data or access via a borrowed institutional device). The only disease-based exclusion criteria are known poor or uncertain prognosis at the time of approach (child not expected to live longer than 6 months). In families with 2 caregivers, both will be eligible to participate in the study, including receiving the intervention and completing study measures; however, 1 caregiver will be asked to identify as the primary participating caregiver at study enrollment.

### Sample Size

Based on the power analyses discussed below, 350 parents and caregivers of children with cancer will be recruited to account for a conservative 25% attrition rate. Of note, although multiple caregivers from each family are eligible to participate, the target sample size of 350 parents and caregivers of children with cancer refers specifically to 350 primary participating caregivers from unique families. Data from nonprimary participating parents will be used only for exploratory analyses. Based on the results from pilot studies, we expect approximately 20% of the 350 families recruited to include a second participating parents and caregivers of children with cancer. Recruitment, retention, and engagement strategies have been an explicit focus of previous eSCCIP research, and strategies generated from this research and the literature inform the recruitment plan [35,36]. Recruitment targets are feasible based on the number of children with cancer treated annually at sites A, B, and C (1000 per year across sites), as well as the number of English-speaking and Spanish-speaking parents and caregivers of children with cancer at each site. We expect 40%-50% of the sample to be Spanish-speaking parents and caregivers of children with cancer. Previous eSCCIP studies have had high recruitment rates (64%-70%) [24,36] as well as retention rates of over 70% in eSCCIP pilot work. Estimating 25% study attrition is an appropriately conservative approach; however, recruitment targets will be evaluated frequently and adjusted accordingly if attrition is higher than expected, given previous work.

To specifically address aim 3, parents and caregivers of children with cancer will be identified from each study site using purposive sampling across demographic groups. We will initially target 10 participants from each site (n=30), comprising 6 parents and caregivers of children with cancer (3 English-speaking and 3 Spanish-speaking), 2 psychosocial providers, and 2 oncology providers, but final recruitment will be dependent upon data saturation. We will complete interviews until no new ideas are shared, consistent with best practices for reaching saturation in qualitative research [37].

### Study Conditions and Delivery

#### eSCCIP Intervention

The content of eSCCIP/eSCCIP-SP has 4 web-based, self-directed cores designed for parents and caregivers of children with cancer to complete over 1 month during their child’s active cancer treatment (including maintenance therapies) or shortly after completion of treatment. Each core is a unique mix of original video content and web-based activities and is complemented by a brief telehealth follow-up with an eSCCIP-trained clinical interventionist (ie, guide). Cores 1-3 include novel content derived directly from evidence-based cognitive behavioral concepts and family system principles modeled from components of the in-person predecessor of eSCCIP; core 4 is designed as a wrap-up core to summarize intervention content. All cores incorporate family systems theory to maintain an interpersonal focus and normalize the family’s experiences. All intervention materials exist in English and Spanish, and telehealth sessions are delivered in English or Spanish, depending on enrollment in eSCCIP or eSCCIP-SP.

Video content falls into two distinct categories: (1) the multifamily video discussion groups, which were initially created for the Surviving Cancer Competently Intervention Program—Newly Diagnosed protocol, consist of recorded and edited conversations between a group of 17 parents and caregivers of children with cancer that serve as a powerful family systems proxy of participants’ interaction with other parents and (2) skill-building videos, where foundational cognitive behavioral intervention skills, such as cognitive reframing, are introduced. The multifamily video discussion groups capture themes common among parents and caregivers of children with cancer (eg, anger, guilt, and emotional exhaustion), examples of coping (eg, putting things in perspective), and the impact on the family over time (eg, siblings, grandparents, and thinking about the future). The skill-building videos feature interviews with parents and providers, as well as graphics and narration illustrating foundational intervention skills. Web-based activities provide opportunities for caregivers to practice the skills they have learned.

All web-based activities are securely captured by the program, allowing the guide to view data entered by participants (eg, free text responses) and integrate participant responses into telehealth follow-up sessions. The telehealth follow-ups after cores 1-3 are the only component of eSCCIP that is scheduled with a trained guide. The telehealth follow-up sessions follow a flexible manualized format to ensure standardization across guides and appropriate delivery of key intervention principles.

#### Education Control

Parents and caregivers of children with cancer randomized to the patient education control condition will be given a unique log-in to access a website with information about psychosocial functioning, coping, and PTSS related to pediatric cancer. This website will be available in both English and Spanish and will consist of information modified from CopingSpace [38], an evidence-informed website developed by Ryan’s Case for Smiles, a national organization dedicated to supporting families impacted by pediatric cancer and other chronic diseases. Topics include age-appropriate explanations and coping strategies for children, the siblings’ unique experience and needs, warning signs a child is not coping well, techniques to reduce your stress and anxiety, and practical tips for managing meals and family needs. Unlike eSCCIP/eSCCIP-SP, the education control website will not include instruction in the cognitive behavioral skills taught in eSCCIP/eSCCIP-SP nor will it include access to the videos or telehealth follow-up sessions in eSCCIP/eSCCIP-SP. The high-quality information provided to parents and caregivers of children with cancer makes this a valuable and meaningful comparator condition.

### Study Platform

The eSCCIP/eSCCIP-SP and education control programs have been built in the most recent version of the Research Infrastructure Containing e-interventions system developed and maintained by the University of Virginia Center for Behavioral Health and Technology. This platform provides the tools to both deliver robust digital health interventions as well as to manage participants in research trials. The system is designed to support projects that contain protected health information and are subsequently subject to compliance with federal and state regulations regarding data of this type. eSCCIP/eSCCIP-SP has been designed to be delivered as a fully automated program, but also it has the functionality to incorporate human support components (eg, facilitate communication between participant and guide). It includes 4 cores and 3 telehealth visits, with the cores automatically made available to the participant over time. After completion of the first core, the first telehealth visit is scheduled for a time in the upcoming week. The second and third cores are available 1 week after the completion of the previous core and include a subsequent telehealth visit. The fourth and final core concludes the intervention with a review of the program. Participants access the intervention through a secured portal, and all data are captured and maintained consistent with Health Insurance Portability and Accountability Act’s guidelines.

### Study Procedure

Potential participants will be screened for eligibility using a brief, web-based screening questionnaire that is accessible by direct contact with a member of the study team or through the study interest website. Once screened, an RC will confirm eligibility and instruct participants about the consent process. Web-based consent procedures will be conducted via DocuSign or a similar secure software. Once web-based consent is complete, participants will receive a unique username and password to log onto their randomized condition (eSCCIP/eSCCIP-SP or education control). Study questionnaires will be completed through this web platform. As there are no required components of the education control, automated or RC-directed text and email reminders to use the program will not be required for individuals randomized to the education control.

After completion of the initial web-based assessment battery, participants in the intervention condition will schedule their initial telehealth session using an automated scheduling tool available through the platform hosting eSCCIP/eSCCIP-SP. Participants will be notified that the overview core and core 1 need to be completed prior to the telehealth session. Automated reminders will be sent at regular intervals to encourage participation in study cores. Manual follow-up by RCs will also be initiated in cores that are not completed. Cores will automatically unlock at 1-week intervals, and telehealth sessions will be scheduled directly with the participant. A total intervention completion window of 4-6 weeks will be anticipated by the study team for each participant, building in some additional time for start-up and potential scheduling delays. A similar process has been successfully implemented in eSCCIP pilot studies [24,30]. Because eSCCIP/eSCCIP-SP cores and the education control website are delivered digitally, all parents and caregivers of children with cancer will have convenient access to their respective condition’s content anywhere the internet is available.

### Treatment Fidelity

The administrative interface of the intervention platform collects several metrics that will be used to record the intervention and monitor participation, including generating a comprehensive activity record of core use (ie, a record of completion of each activity and a record of all free-response answers). Fidelity checklists and session outlines have also been developed for the telehealth follow-up sessions. The designated Fidelity Consultant, a pediatric psychologist with extensive experience delivering psychosocial intervention to families, will watch and code 15% of all telehealth sessions for each guide using the fidelity checklists as a measure of treatment fidelity to strengthen study rigor.

### Study Outcomes

Data will be collected at 3 time points: prior to beginning the intervention (T1), postintervention (after 6 weeks; T2), and at 3-month follow-up (3 months after baseline; T3). Outcome measures include the Psychosocial Assessment Tool (PAT) [39,40], the Multidimensional Scale of Perceived Social Support [41], the Kessler Psychological Distress Scale (K-6) [42,43], the COVID-19 Exposure and Family Impact Scale [44], the Coping Self-Efficacy Scale [45], the Cognitive Emotion Regulation Questionnaire [46], Distress Thermometer [47], the Patient-Reported Outcomes Measurement Information System Short Form (PROMIS Short Form v1.0—Anxiety-8a) [48], the posttraumatic stress disorder checklist [49], as data on previous psychosocial service usage. Individuals randomly assigned to the intervention condition will also complete a questionnaire at T3 measuring satisfaction and acceptability with the intervention, which has been used in all prior eSCCIP studies. Measure description and schedule for data acquisition are provided in Table 1.

**Table 1:** Outcome measures and assessment timing schedule.

Domain or measure	Description	T1^a^	T2^b^	T3^c^
Chart review	Review of patient medical record			
Demographic variables of parents and caregivers of children with cancer	Demographic variables of enrolled parents and caregivers of children with cancer	✓		
Previous psychosocial service usage	Questionnaire to measure contact and work with social work, psychology, psychiatry, pastoral care, community, and hospital-based services for all members of the family	✓	✓	✓
PAT^d^	Brief parent report screener of psychosocial risk [39,40]	✓		
MSPSS^e^	Questionnaire to measure perceived social support from family, friends, and significant other [41]	✓	✓	✓
K-6^f^	Questionnaire to measure acute distress [42,43]	✓	✓	✓
CEFIS^g^	Questionnaire to measure the extent to which a family has experienced or been “exposed” to COVID-19–related potentially traumatic events (eg, economic changes, illness in family) and the impact of these events on the family’s functioning and well-being [44]	✓	✓	✓
CSES^h^	Questionnaire to measure the perceived ability to cope effectively with challenging situations across 3 domains (use problem-focused coping, stop unpleasant emotions and thoughts, and get support from friends and family) [45]	✓	✓	✓
CERQ^i^	Questionnaire to identify cognitive coping strategies used after a negative event or situation. Measures of 9 different cognitive coping strategies [46]	✓	✓	✓
Distress thermometer	Single-item indicator of psychosocial distress that is widely used in the oncology literature [47]	✓	✓	✓
PROMIS^j^ Short Form v1.0—Anxiety-8a	Questionnaire measuring symptoms of anxiety [48]	✓	✓	✓
PCL-5^k^	Questionnaire to assess for symptoms of posttraumatic stress disorder in the civilian population [49]	✓	✓	✓
eSCCIP^l^ Evaluation Questionnaire	Questionnaire to measure intervention acceptability		✓	✓

^a^T1: preintervention (baseline).

^b^T2: postintervention (after 6 weeks).

^c^T3: follow-up (3 months after baseline).

^d^PAT: Psychosocial Assessment Tool.

^e^MSPSS: Multidimensional Scale of Perceived Social Support.

^f^K-6: Kessler Psychological Distress Scale.

^g^CEFIS: COVID-19 Exposure and Family Impact Scale.

^h^CSES: Coping Self-Efficacy Scale.

^i^CERQ: Cognitive Emotion Regulation Questionnaire.

^j^PROMIS: Patient-Reported Outcomes Measurement Information System.

^k^PCL-5: posttraumatic stress disorder checklist.

^l^eSCCIP: Electronic Surviving Cancer Competently Intervention Program.

### Power Analyses and Sample Size Consideration

The primary end point of the study is to compare posttest differences in distress post intervention between intervention and education control groups. In a recent pilot study of 14 participants, we detected a mean change of 1.71 (SD 2.09) in distress on the distress thermometer at 1 month in the English version of the questionnaires. In standardized form, the effect size is 0.82. We do not have an estimate of the impact of the education control group on our primary end point; however, we expect no more than a modest to medium effect (0.2 to 0.5). Therefore, we are planning a sample size to detect a difference in effects between the groups of at least 0.3. Power analyses based on the analysis of covariance (ANCOVA) model and assuming a correlation of at least 0.5 between pretest and posttest scores suggest that a sample size of 350 individual parents and caregivers of children with cancer (175 in each group) can detect a difference of ≥0.3 in standardized mean distress reduction between intervention and education group conditions with a power of at least 80% at the level of significance of .05 and assuming an attrition rate of 25%. Therefore, a sample size of 350 is planned for this study.

### Data Quality

Data quality will be monitored throughout intervention implementation and data collection. The research team will routinely generate data quality reports (via built-in functionality) and meet biweekly to review the reports, discuss any identified issues (eg, missing data), and outline remedial actions. For the education control website, data from CopingSpace.org will be migrated to the same server that will host eSCCIP/eSCCIP-SP for ease of tracking and standardization. Once data collection is complete, a rigorous data cleaning, description, and covariate screening process will be carried out prior to analysis. Data will be first inspected for erroneous, outlying, or potentially high-leverage values, and missing data patterns tabulated and visualized. Data from the 3 recruiting study sites will then be pooled for analyses in 2 different versions of the data set separating participants who provide data for at least 1 measurement visit from participants who provide data at all time points. Multiple imputations will be used to handle missing data, including determination of a primary missing data mechanism, appropriate selection of auxiliary variables (if applicable), and sensitivity analyses to check for robustness of underlying assumptions (eg, MNAR selection models, pattern mixture models) [50]. Efficacy analyses will be carried out for both sets, thus providing a complete picture of whether and how missing data impact analysis results.

### Planned Statistical Analyses

#### Aim 1: Analyses

The primary end point of specific aim 1 is the change in acute distress as measured by the K-6 distress scale over 1 month (immediately post intervention). To test whether eSCCIP/eSCCIP-SP is more efficacious in decreasing acute distress relative to the education control (hypothesis 1), an ANCOVA model will be estimated, which has repeatedly been shown to provide optimal estimate precision, power, and CI coverage among pretest-posttest analysis alternatives in RCTs [51,52]. The ANCOVA model will be used to test for group differences in K-6 distress scores, controlling for preintervention distress scores. Covariates will be incorporated if baseline differences are identified across site-specific, program delivery, and demographic variables. As participants may be nested within providers at each site, we will explore the need for specifying a mixed-effects model, contingent upon the size of the provider effect (eg, design effect, impact on model standard errors) and the number of providers available in the data set. With regard to families with 2 participating parents and caregivers of children with cancer, methodological research has shown poor model performance of mixed-effects dyadic models if the proportion of singletons (dyads with only 1 member) is high, as is expected for this study; therefore, primary analyses will be conducted only on primary participating caregivers [53]. We do not expect treatment effects to differ by dyad status and will test this assumption with data obtained from nonprimary participating parents analyzed in exploratory analyses. Model assumptions will be checked and alternative robust will be methods used, for instance, robust SEs, bootstrapping, or generalized linear models, if applicable [54,55]. Secondary end points (eg, PTSS and anxiety) in specific aim 1 will be analyzed in the same manner. All tests will be 2-tailed at a .05 level of significance; the Benjamini-Hochberg method will be used to adjust for multiple testing [56].

#### Aim 2: Analyses

Specific aim 2 analyses will further probe the eSCCIP/eSCCIP-SP user experience, intervention effects, and boundary conditions. First, descriptive summaries and data visualizations will be used to explore user engagement variables, such as the number of cores completed. Next, potential risk and protective factors will be examined by introducing interaction effects to the models previously described for specific aim 1, including all primary and secondary end points. Interaction terms will be formed between intervention assignment and each potential moderator variable, tested in separate models under the Benjamini-Hochberg control method. Follow-up inferential and graphical procedures will be used to interpret significant interaction effects [57]. Variables selected as potential moderators are participant characteristics guided by the Behavior Change Model for Internet Interventions (eg, race, gender, and socioeconomic status) and family risk variables guided by the Pediatric Preventative Health Model (eg, COVID-19 Exposure and Family Impact Scale score, PAT baseline score). Of primary importance is the potential moderating effect of intervention language, which will similarly be tested. Additional exploratory analyses will be carried out to answer key auxiliary questions and maximize the use of the data collected. First, we will examine any possible persisting effects of eSCCIP/eSCCIP-SP at the 3-month follow-up time point, based on longitudinal analysis models designed for randomized pretest-posttest-follow-up designs [58]. Similarly, we will also explore whether treatment effects differed between parents and caregivers of children with cancer from families with 1 or 2 participating caregivers, as well as examine trends in data provided by nonprimary participating caregivers.

#### Aim 3: Analyses

Qualitative interviews will be coded and analyzed using the CFIR framework, a widely used implementation science framework. An initial codebook will be generated using different domains of the CFIR framework, focusing on the constructs within each domain that are most related to the implementation of eSCCIP/eSCCIP-SP (eg, adaptability, cost, patient needs and resources, and self-efficacy). Discrepancies will be identified and resolved by consensus among the investigators. To strengthen the rigor of the analysis, a primary and secondary coder will be assigned as the analysis continues with the secondary coder checking the coding of the primary coder as well as completing a summary of data on each category within that case (group) and across all cases (groups). The case summaries and samples of the coding process will be reviewed in regular meetings of the study team. Through this content analysis process, codes will be transformed into categories and categories into themes according to national standards that identify and refine strategies for the implementation of the intervention. Themes that result from this process will be translated into implementation and sustainability strategies organized around the 5 major domains of the CFIR framework. We will explore intervention characteristics, inner and outer settings, characteristics of individuals, and the implementation process.

The incremental (ie, beyond the cost of an education control website) costs and cost-savings analysis will follow best practices. We will estimate incremental costs associated with eSCCIP/eSCCIP-SP treatment, costs per participant, and marginal costs per unit of acute distress reduction. All costs will be estimated and evaluated in constant dollars using the appropriate index for price adjustment. Those resources that rise at the rate of general inflation will be corrected for inflation with the consumer price index, while medical costs will be adjusted with the medical component of the consumer price index. Due to the efficacy trial nature of this project, we will conduct a partial economic evaluation to document the cost information of the eSCCIP/eSCCIP-SP and estimate a preliminary cost-effectiveness regarding the primary outcome (ie, acute distress reduction). We will evaluate the intervention costs from health care provider perspective; therefore, the nonresearch-related cost/resource will be those health care provider–related ones, which will include intervention labor costs for hourly wages including fringe benefits of delivery staff for a different component of the program (ie, website regular maintenance staff, and telehealth guide). During all phases of development and implementation, we will document what was performed, by whom, the length of execution, and the use of any nonhuman resources. Cost collection instruments will be modified based on the eSCCIP guide/research team templates and behavioral intervention templates. Care will be taken to accurately separate research-based costs (eg, costs of follow-up assessments) from actual worksite program implementation costs. We will estimate separate costs for eSCCIP and eSCCIP-SP to provide the information needed to compare differences and provide evidence for predicting costs based on patients’ ethnicity distribution. To capture cost savings from a health care provider’s perspective, we will use electronic patient record to track control group patients’ related health care service usage, which provides the relevant information to estimate potential cost savings patients in the treatment group could have used if there is no eSCCIP/eSCCIP-SP. A full-scale cost-effectiveness analysis will be out of the scope of this efficacy study. We will calculate incremental cost-effectiveness ratios and will use bootstrapping methods to account for the uncertainty around the incremental cost-effectiveness ratios but recognize that the limited scope of this study: only collecting partial information on program costs, especially on implementation costs [59]. We will calculate net benefits, benefit-cost ratio, and return on investment (return on investment=net benefits/costs×100). Bootstrapping will be used for CIs construction around those measures. Generalized linear models will be applied to estimate the relationship between program incremental costs and the characteristics of participants, providers, and programs. Appropriate link function and distribution family will be chosen to fit the data. A 2-part model will be used to examine and handle sample selection and nonrandom attrition.

## Results

This RCT was funded by the National Cancer Institute (1R01CA258668) in March 2022. Recruitment is planned to begin by February 2023 and will continue for 3 years.

## Discussion

### Principal Findings

This study protocol details an RCT to evaluate the psychosocial efficacy of eSCCIP/eSCCIP-SP, a novel digital health intervention for parents and caregivers of children with cancer. The development of eSCCIP and eSCCIP-SP was thoughtfully guided by the psychology, culturally competent care, and technology literature. Considering the recent and ongoing COVID-19 pandemic, the need for flexible, acceptable, and accessible psychosocial digital health intervention is more apparent than ever before to provide evidence-based psychosocial care for parents and caregivers of children with cancer. We hypothesize that the intervention will be more efficacious than an education control at decreasing negative symptoms of acute distress, anxiety, and PTSS symptoms while demonstrating improvements to coping self-efficacy and positive cognitive coping strategies.

### Comparison With Prior Work

Despite the well-documented and established psychosocial needs of parents and caregivers of children with cancer as well as challenges to in-person engagement, exceedingly few digital health options have been evaluated to provide care to this population. To our knowledge, the web-based adaptation of the Bright IDEAS Maternal Problem-Solving Skills Training Intervention [16,33,34] is the only existent psychosocial digital health intervention for parents and caregivers of children with cancer. This intervention provides education to improve problem-solving skills.

The proposed study is innovative for several key reasons. The availability of an evidence-based, psychosocial digital health intervention for parents and caregivers of children with cancer available in English and Spanish is novel, especially given its hybrid format of including both self-guided web-based content and live telehealth sessions with a trained interventionist. This format capitalizes on recommendations from prior electronic adaptations of manualized psychosocial support for parents and caregivers of children with cancer, which has suggested that web-based content may be most effective when combined with in-person contact and support [33]. The eSCCIP/eSCCIP-SP intervention has been rigorously designed and developed and is now ready for large-scale testing. Second, the development of eSCCIP/eSCCIP-SP used a systematic participatory process with multiple groups of stakeholders at all stages of design and initial testing, increasing the likelihood that the intervention is responsive to participant needs and reflects the “true” needs of the target population. In addition to working closely with parents and caregivers of children with cancer, we also partnered with trained medical interpreters and experts in cultural intervention adaptation when developing the eSCCIP-SP. Although this type of participatory research is increasingly important and well used in social and behavioral research, it is not yet commonplace in pediatric psychosocial oncology and further serves to distinguish our tools and approach. Third, the eSCCIP-SP is innovative, given the lack of resources available to the high-risk population of parents and caregivers of children with cancer, who are of Latinx descent, and the potential of the intervention to reduce systemic health care disparities related to psychosocial services. Fourth, eSCCIP/eSCCIP-SP can be used anywhere with an internet connection (including cellular telephone data access or Wi-Fi) and is adaptable/responsive to devices (eg, mobile phones), decreasing barriers to access and making the intervention highly amenable to eventual broad and rapid dissemination. While delivering web-based psychosocial interventions itself is not altogether novel, these interventions are largely unavailable for parents and caregivers of children with cancer. This is especially timely given the recent rise and acceptability of telehealth and video platforms for health care service delivery during the COVID-19 pandemic. Fifth, we will use an advanced platform to closely track engagement metrics, which will allow us to develop and quickly implement stepped care plans for future implementation studies. This platform has been developed by the Center for Behavioral Health and Technology at the University of Virginia and is used successfully to manage numerous behavioral and psychosocial digital health intervention trials, including large studies in adult oncology and adolescent oncology [60-62].

### Limitations

It is possible that the sample of parents and caregivers of children with cancer who opt to participate in our research are self-selected and may not fully represent the population of caregiving individuals of children with cancer. To identify and more broadly implement successful recruitment, retention, and engagement strategies, the primary study team recently published a manuscript describing qualitative data from focus groups and interviews [36]. The sample included past eSCCIP participants and parents and caregivers of children with cancer who were naïve to eSCCIP (but viewed some intervention content prior to participating). By implementing these strategies broadly over the course of the study, a wide range of potentially eligible parents and caregivers of children with cancer will be targeted for inclusion. We will monitor recruitment and enrollment rates closely over the course of the study, adjusting recruitment strategies if indicated. It is also possible, given both the duration of the study follow-up and randomization procedures, that participants may drop out or be lost to follow-up. Efforts by research staff to maintain participation in the study and to obtain postintervention measures for all participants will be made, including participants providing multiple means of contact to aid in follow-up as well as receiving study payments after completion of each data collection time point. In eSCCIP development trials, close tracking and monitoring procedures led to high retention rates for participants who begin the intervention, and the addition of automated reminders and a stepped care approach to communicate with participants will bolster these efforts. The characteristics of completers versus noncompleters will also be examined for systematic differences, and we will use an intent-to-treat design for data analyses.

Second, since the intervention involves an active intervention component, including telehealth follow-up with a trained guide for participants allocated to eSCCIP/eSCCIP-SP, blinding of guides is not possible. However, study team members not involved in intervention delivery will not have access to random assignment information. After randomization, participants will be instructed on the procedures within their assigned group. All outcome measures will be completed directly on the internet by study participants, further reducing the risk of bias.

Finally, it is also important to acknowledge that care at study sites includes access to psychosocial support; however, the use of these services is likely to be equivalent across intervention and control group participants, and no participant in either condition will be discouraged from accessing any available psychosocial support. While usual care may include work with a licensed psychologist, novel aspects of eSCCIP are unique to the intervention and not a component of routine care. We will also collect information on mental and psychosocial health services used by all participants during the study period to understand the context in which eSCCIP/eSCCIP-SP was received and control for other psychosocial services in analyses if indicated.

### Dissemination Plan

Data will be disseminated and shared with the scientific community through peer-reviewed publications and academic conference presentations. Furthermore, the results will be shared with participants through annual updates. Continual efforts to disseminate study progress and results are valued to maintain productive and collaborative relationships with institutional care teams and relevant stakeholders. A summary of study results will be submitted to ClinicalTrials.gov within 1 year of the trial’s primary completion date.

### Conclusions

Despite advances and improved medical outcomes for childhood cancer, parents and caregivers of children with cancer are at known risk for negative psychosocial sequelae, including increased risk for Latinx, Spanish-speaking parents and caregivers of children with cancer [1,2,12]. Established standards of care recommend access to high-quality psychosocial support for parents and caregivers of children with cancer and, while digital health interventions may address many logistical barriers to in-person participation in this population [63], few options exist at this time. The results from rigorous development, refinement, and pilot testing of eSCCIP and eSCCIP-SP have demonstrated the potential to deliver an effective and easily disseminatable intervention, decreasing barriers to care, and improving psychosocial outcomes. Data obtained from this RCT will be instrumental in evaluating the efficacy of the eSCCIP/eSCCIP-SP intervention as an evidence-based tool to support parents and caregivers of children with cancer across the cancer trajectory.

## Abbreviations

ANCOVA: analysis of covariance
CFIR: Consolidated Framework for Implementation Research
eSCCIP: Electronic Surviving Cancer Competently Intervention Program
eSCCIP-SP: El Programa Electronico de Intervencion para Superar Cancer Competentemente
K-6: Kessler Psychological Distress Scale
PAT: Psychosocial Assessment Tool
PROMIS: Patient-Reported Outcomes Measurement Information System
PTSS: posttraumatic stress symptom
RC: research coordinator
RCT: randomized controlled trial

## Data Availability

We would be happy to share deidentified study data upon request with other research groups who request access or valid research purposes. Dissemination of study results to the scientific and lay community is described in the Dissemination Plan subsection of the main manuscript text.
